# Expression of Human ACE2 N-terminal Domain, Part of the Receptor for SARS-CoV-2, in Fusion With Maltose-Binding Protein, *E. coli* Ribonuclease I and Human RNase A

**DOI:** 10.3389/fmicb.2021.660149

**Published:** 2021-06-11

**Authors:** Shuang-yong Xu, Alexey Fomenkov, Tien-Hao Chen, Erbay Yigit

**Affiliations:** New England Biolabs, Inc., Ipswich, MA, United States

**Keywords:** *E. coli* ribonuclease I (RNase I), RNase III, RNase I-ACE2NTD fusion, human RNase A-ACE2NTD fusion, SARS-CoV-2, human ACE2 receptor

## Abstract

The SARS-CoV-2 viral genome contains a positive-strand single-stranded RNA of ∼30 kb. Human ACE2 protein is the receptor for SARS-CoV-2 virus attachment and infection. We propose to use ribonucleases (RNases) as antiviral agents to destroy the viral genome *in vitro*. In the virions, the RNA is protected by viral capsid proteins, membrane proteins, and nucleocapsid proteins. To utilize RNases as antiviral strategy, we set out to construct RNase fusion with human ACE2 receptor N-terminal domain (ACE2NTD). We expressed six proteins in *E. coli* cells: (1) MBP-ACE2NTD, (2) ACE2NTD-GFP, (3) RNase I (6×His), (4) RNase III (6×His), (5) RNase I-ACE2NTD (6×His), and (6) human RNase A-ACE2NTD (6×His). We evaluated fusion expression in different *E. coli* strains, partially purified MBP-ACE2NTD protein from the soluble fraction of bacterial cell lysate, and refolded MBP-ACE2NTD protein from inclusion body. The engineered RNase I-ACE2NTD (6×His) and hRNase A-ACE2NTD (6×His) fusions are active in cleaving SARS-CoV-2 RNA fragment *in vitro*. The recombinant RNase I (6×His) and RNase III (6×His) are active in cleaving RNA and dsRNA in test tube. This study provides a proof-of-concept for construction of fusion protein between human receptor and nuclease that may be used to degrade viral nucleic acids.

## Graphical Abstract

Cartoon illustration part of this work (Human ACE2 N-terminal domain tethered to RNase A and RNA degradation by the fusion enzyme).

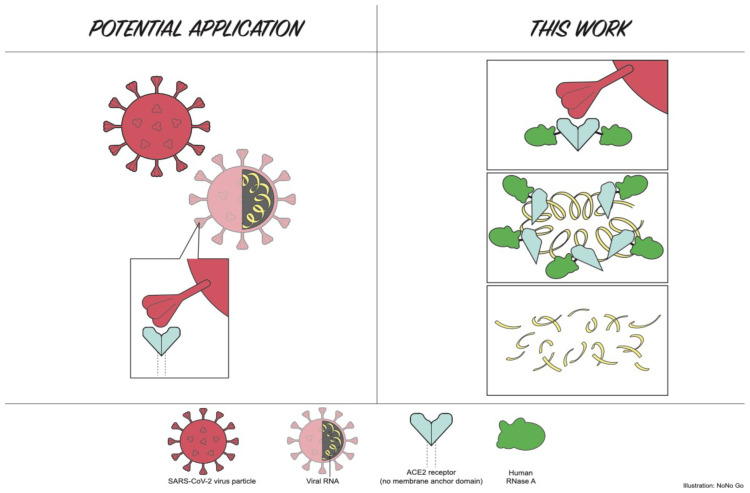

## Introduction

Human angiotensin-converting enzyme 2 (ACE2 or ACE-2) is a carboxypeptidase, which converts angiotensin I to angiotensins 1–9, and it catalyzes the degradation of angiotensin II to angiotensins 1–7 [reviewed in Gaddam et al. (2014) and Santos et al. (2019)], which attenuates inflammation and oxidative stress caused by angiotensin II. ACE2 is a glycoprotein that serves as host cell receptor for the severe acute respiratory syndrome coronavirus (SARS-CoV), human coronavirus NL63/HCoV-NL63, and SARS-CoV-2 (Li et al., 2003; Hoffmann et al., 2020). The SARS-CoV-2 is currently causing pandemics and COVID-19 respiratory disease around the globe since its initial outbreak in Wuhan, Hubei Province of China in December 2019 (Wang et al., 2020). The first genome sequence was deposited in GenBank/NCBI on January 13, 2020 (Wuhan seafood market pneumonia virus isolate Wuhan-Hu-1, GenBank accession number NC_045512). The mortality rate among the elderly with underlying conditions is relatively high, while the overall fatality rate from positively diagnosed patients in the world stands at ∼6.26% as of April 14, 2020 (120,863 deaths among over 1.9 million infected when this work was half-finished) (Bar-On et al., 2020; Li et al., 2020). A year later the total number of global cases have reached 136 million with a death toll of 2.94 million as of April 12, 2021 (∼2.2% uncorrected raw case mortality rate). For comparison, the global mortality associated with seasonal influenza epidemics from 2002 to 2011 (no data in 2009) from the World Health Organization was estimated at 0.00058%, or approximately 6 in 10,000 infected individuals in all age groups (Paget et al., 2019). Thus, COVID-19 is approximately 10–100 times more lethal in causing human death than seasonal flu, although the mortality rate varies from country to country depending on the medical infrastructure. In majority of COVID-19-positive cases (estimated at ∼70 to 80%), the infected individuals show symptoms with fever and chill, severe muscle pain, cough, symptoms of upper respiratory tract infection and will likely recover after 2–3 weeks. However, the other 20–30% patients require hospitalization, and roughly 5% patients become critically ill and require intensive care, and some would eventually develop severe pneumonia (acute respiratory distress syndrome), cardiovascular damage, acute kidney injury, and catastrophic organ failure, possibly owing to acute cytokine storm of massive host immunological reactions (Huang et al., 2020; Lake, 2020; Mehta et al., 2020). The percentage of asymptomatic positive carriers is unknown due to the lack of extensive antibody testing in the general population. The soluble recombinant human ACE2 protein has been shown to drastically reduce virus titer in cell cultures by binding to SARS-CoV-2 virus and preventing reinfection (Monteil et al., 2020).

The SARS viral Spike (S) protein mediates virus entry into host cells by first binding to the ACE2 receptor through the receptor-binding domain (RBD) in the N-terminal S1 domain (the trimeric Spike protein also binds cellular heparin). Following protease cleavage, fusion of the viral and host membranes through the S2 domain is ensued by endocytosis and subsequent release of viral RNA for translation of viral proteins, post-translational processing, viral RNA replication and modification, and virus particle maturation in the Golgi apparatus (Tripet et al., 2004; Wong et al., 2004; Wang et al., 2008; Millet and Whittaker, 2018). The life cycle of SARS-CoV-2 is probably very similar to that of SARS-CoV (Hoffmann et al., 2020; Ou et al., 2020). The receptor-binding domain of the S protein (S-RBD) serves as a molecular target for developing virus attachment inhibitors, therapeutic antibodies, and preventive vaccines (Du et al., 2009). The structures of ACE2 receptor with bound S1 domain or full-length S protein of SARS-CoV-2 have been solved recently at high resolution by Cryo-EM (Wrapp et al., 2020; Yan et al., 2020). Recently, COVID-19 virus variants have been isolated in the United Kingdom, South Africa, Brazil, India, and the United States that contain multiple amino acid (aa) mutations in the S protein (e.g., single mutant K417N, E484K, N501Y; double mutant L452R/E484Q, ΔH69/ΔV70 deletion mutant, and variants with multiple mutations) that may have increased the binding affinity to human ACE2 receptor, with more efficient membrane fusion capability (S2), and overall enhanced transmission rate (Kemp et al., 2020). Another host protein, the transmembrane protease, serine 2 (TMPRSS2) serves to cleave the SARS-CoV-2 Spike protein to facilitate cellular attachment and membrane fusion (Hoffmann et al., 2020),while other cellular protease furin or cathepsin B/L proteases can also cleave S protein and activate the virus for reinfection of other cells. The full-length TMPRSS2 protein is 492-aa long, that can be cleaved into two polypeptides by autocatalysis: N-terminal non-catalytic domain (aa 1–255), and C-terminal catalytic domain (aa 256–492). The serine protease facilitates the phylogenetically related SARS-CoV virus–cell membrane fusions by proteolytically cleaving and processing the viral Spike glycoproteins (Glowacka et al., 2011). Over-expression of TMPRSS2 in human VeroE6 cells resulted in a cell line VeroE6/TMPRSS2 highly susceptible to SARS-CoV-2 infection and potentially useful for generating high virus titers (Matsuyama et al., 2020). TMPRSS2 is also a therapeutic target for drug development to impair virus–cell attachment and membrane fusion.

Current strategies to reduce viral transmission among individuals are social distancing (quarantine and/or mandatory shelter in place/locked down) to reduce community transmission, chemical treatment, and sanitization of public places that may harbor the viral particles, creation of physical barriers (wearing masks, facial shields, protective goggles, and gowns). The SARS-CoV particles on smooth surfaces retained their viability for over 5 days at 22–25°C and relative humidity of 40–50%; however, virus viability was rapidly lost at higher temperatures (>38°C) and higher relative humidity (Chan et al., 2011). The survival of infectious SARS-CoV-2 virus particle in air droplets and solid surfaces is estimated in a few hours to days (van Doremalen et al., 2020). Virus particles in the respiratory air droplets (aerosol) from asymptomatic carriers and patients are suspected as transmission route, which resulted in high transmission rate to health care workers in hospitals and social gathering events. The degradation and loss of infectivity of the virus particles relies on natural decays of the infectious particles possibly by many environmental factors such as temperature, humidity, pH, protease and RNase, and other natural microbial enzymes present under environmental settings. Four vaccines against SARS-CoV-2 Spike (S) protein have been developed by pharmaceutical companies and are being applied to frontline medical professionals, senior citizens, essential workers, and later to the general population (Ahmed et al., 2020; Anderson et al., 2020; Jackson et al., 2020a, b; Mulligan et al., 2020; Walsh et al., 2020; Dai and Gao, 2021; Knoll and Wonodi, 2021; Sadoff et al., 2021).

Clinical trials have been carried out for the treatment of COVID-19 with the nucleotide analog RNA-dependent RNA polymerase (RdRP) inhibitor Remdesivir^®^, which has been approved by the U.S. Food and Drug Administration to treat COVID-19 patients. Other antiviral drugs include protease inhibitors that inhibit the viral protease (main protease, Mpro) required for the processing of virus polyprotein into functional enzymes (RdRP, RNA helicase, and ribonuclease) for viral genome replication. Other host factors involved in viral replication and maturations have been identified and are being targeted for therapeutic intervention (Gordon et al., 2020). The overall economic fallout from the SARS-CoV-2 pandemics in the global economy is difficult to quantitate, but the pandemics had negatively impacted on economic activities and GDP, leading to a global recession in 2020.

The *E. coli* MBP protein expression system utilizes a strong promoter for transcription and a strong ribosome binding site for protein synthesis, and the production of target protein is inducible by addition of IPTG^1^ (Riggs, 2000). Some MBP fusion proteins can be expressed at high level, up to 100 mg per liter (L) of induced cells. In addition, the target protein in fusion with MBP can be exported into bacterial periplasm to avoid toxicity since over-expression of certain enzymes in cytoplasm is toxic to the host. The MBP signal peptide directed the fusion protein to the periplasm with cleavage of MBP signal peptide. In some cases, MBP fusion with the target protein is active, and there is no need to cleave off the MBP tag for enzymatic activity [e.g., MBP-PNGase F (NEB Catalog 2019/20), RNase I_f_ (MBP-RNase I fusion), and MBP-*Geobacillus* intron-encoded reverse transcriptase (SYX, unpublished)]. The MBP tag can also serve as a chaperon to enhance the fusion protein solubility (Riggs, 2000; Nallamsetty and Waugh, 2006). The *E. coli* ribonuclease I (RNase I, periplasmic isoform) belongs to the RNase T2/S-RNase group of endoribonucleases, and it had been over-expressed and purified (Padmanabhan et al., 2001). Bovine RNase A and *E. coli* RNase I, commercially available in research grade for many years, are widely used to eliminate RNA in the preparation of plasmid and genomic DNA for molecular biology applications (Bechhofer and Deutscher, 2019). *E. coli* RNase III is an endoribonuclease-cleaving double-stranded (ds) duplex RNA that is involved in transcript degradation and accelerated RNA decay, and it requires divalent cations for activity (Bechhofer and Deutscher, 2019). RNase III and its variants can generate small dsRNA (21–23 bp) in Mn^2+^ buffer to be used in small RNA-medicated gene transcription regulation (Xiao et al., 2009). The human ribonuclease A (hRNase A) has eight different secreted variants (isoforms) that have antiviral/antibacterial/antifungal activities and cytotoxicity against intracellular parasites. hRNase A (pancreatic) encoded by the human *rnase1* gene is an endoribonuclease cleaving the 3′ side of pyrimidine nucleotides of single-stranded (ss) and double-stranded (ds) RNA (Sorrentino et al., 2003; Koczera et al., 2016). The hRNase A precursor contains 156 aa residues with a signal peptide of 28 residues (128 aa in mature protein, Uniprot ID-P07998). The potential of hRNase A as an anti-Covid-19 reagent has not been extensively explored. Host-encoded defense ribonucleases as anti-viral agents against ssRNA virus genomes have been reviewed recently (Li and Boix, 2021). A previous study has shown that the protective function of RNase L *in vivo* (activated dimer) against a mouse coronavirus (MHV-JHM strain) is associated with cell type specific and regional restriction of viral replication in the gray matter and ameliorated neurodegeneration and demyelination (Ireland et al., 2009).

The SARS-CoV-2 viral genome is a positive-strand ssRNA of ∼30 kb possibly encoding at least 29 viral proteins (Lu et al., 2020) [reviewed in Bar-On et al. (2020)]. In this study, we propose to use ribonucleases (RNases) as an antiviral agent to destroy the viral genome. Our final goal is to construct RNase I fusion with human ACE2 receptor protein and to investigate strategies to trigger the destabilization of the viral particles or partially release the RNA genome to become more accessible to RNase degradation. To achieve this goal as a first step, we expressed six proteins in *E. coli* cells: (1) MBP-ACE2NTD, (2) ACE2 NTD-GFP, (3) RNase I (6×His), (4) RNase III (6×His), (5) RNase I-ACE2NTD (6×His), (6) hRNase A-ACE2NTD. We examined expression conditions and partially purified the fusion protein MBP-ACE2NTD. We optimized expression conditions and strains for RNase I (6×His) and RNase III (6×His). We also constructed and partially purified the fusion enzyme RNase I-ACE2NTD (6×His), which is active in cleaving RNA in the absence of divalent cations (EDTA resistant). We also demonstrated that hRNase A-ACE2NTD (6×His) fusion is active in the digestion of SARS-CoV-2 RNA fragment. We fused ACE2-NTD to GFP that resulted in reduced green fluorescence signal. This construct may be useful to isolate more soluble variants of ACE2NTD by screening mutant libraries. This work serves as a proof of concept for the construction of artificial ribonuclease fused to human ACE2 receptor NTD. The availability of inexpensive recombinant ACE2NTD protein may facilitate the screening of small molecules binding to ACE2 and block SARS virus attachment and entry.

## Materials and Methods

### *Escherichia coli* Strains and Recombinant DNA Method

*E. coli* strains for fusion protein expression are NEB Express (B strain), NEB Turbo (K strain), NEB SHuffle^®^T7 (K and B strains), and Nico (λDE3) derived from BL21. The periplasm expression vector pMAL-p5× (Amp^R^) was used for the construction of ACE2NTD fusion. Synthetic gene blocks (gblock) with optimized *E. coli* codons were purchased from Integrated DNA Technology (IDT) and cloned into the expression vector (*Nde*I and *Bam*HI digested) by NEB HiFi assembly enzyme mix (E2621S, NEB) according to the manufacturer’s instruction. Assembled vector with potential insert was transferred into *E. coli* competent cells by transformation. Individual Amp^R^ transformants were picked and cultured in 2 ml of LB + Amp and shaken at 37°C for 5–6 h. Cells (1.5 ml) were spun down and kept in −20°C for plasmid mini-preparation until needed. LB + Amp (10 ml) was added to the remaining cells and cultured for ∼2–3 h to reach the late log phase. IPTG at 0.3 mM final concentration was added for fusion protein production overnight (at 16 to 18°C). Plasmid DNA was prepared by a Monarch^®^mini-preparation kit (NEB). The inserts were sequenced by two primers [forward primer S1273 from the C-terminal coding region of MBP, and a reverse primer (R2913) downstream of the *Bam*HI site of pMAL-p5×] with a BigDye^®^terminator V3.1 cycle sequencing kit (ABI/Thermo-Fisher). Amylose resin (E8021S, NEB) and amylose column chromatography was carried out according to the manufacturer’s protocol. Protein refolding kit was purchased from Novagen. Proteins in pellet (inclusion body) after centrifugation (10,000 × *g*) was resuspended in a solubilization buffer (10 mM CAPS, pH 11.0, 0.3% N-lauroylsarcosine) and pelleted in a microcentrifuge (4°C). After washing the pellet with wash buffer twice (20 mM Tris–HCl, pH 7.5, 10 mM EDTA, 1% Triton X-100), the pellet was solubilized and dialyzed against 2 L of dialysis buffer (20 mM Tris–HCl, pH 8.5, 1 mM DTT) for 4 h to overnight at 4°C with exchange of the dialysis buffer twice. A small fraction of the refolded MBP-ACE2NTD fusion protein was further purified by amylose magnetic beads (NEB) according to the protocol provided by the manufacturer.

### Description of the Amino Acid Sequence of MBP-ACE2NTD Fusion

The full-length ACE2 protein has 805 aa residues (GenBank sequence ID: BAB40370.1). The first 17 aa residues form the signal peptide not required for activity; the extracellular domain consisted of aa residues 18–740 (Lubbe et al., 2020). Therefore, only the coding sequence for aa residues 12 to 444 is synthesized in two gene blocks (IDT) (the first 11 aa residues were deleted, #12 Val codon was changed to ATG codon for cloning purpose. See the modified protein sequence below). The S2 domain involved in membrane fusion is not included here.

### Maltose Binding Protein (MBP)-ACE2NTD (*Nde*I)

MSSSSWLLLS LMAVTAAQST IEEQAKTFLD KFNHEAEDLFYQSSLASWNY

NTNITEENVQ NMNNAGDKWS AFLKEQSTLA QMYPLQEIQN LTVKLQLQAL

QQNGSSVLSE DKSKRLNTIL NTMSTIYSTG KVCNPDNPQE CLLLEPGLNE

IMANSLDYNE RLWAWESWRS EVGKQLRPLY EEYVVLKNEM ARANHYEDYG

DYWRGDYEVN GVDGYDYSRG QLIEDVEHTF EEIKPLYEHL HAYVRAKLMN

AYPSYISPIG CLPAHLLGDM WGRFWTNLYS LTVPFGQKPN IDVTDAMVDQ

AWDAQRIFKE AEKFFVSVGL PNMTQGFWEN SMLTDPGNVQ KAVCHPTAWD

LG*K*GDFRILM CTKVTMDDFL TAHHEMGHIQ YDMAYAAQPF LLRNGANEGF

HEAVGEIMSL SAATPKHLKS IGLLSPDFQE DNETEINFLL KQAL (*Bam*HI)

(The aa residues for Spike protein binding are underlined).

The codon-optimized gblocks were assembled into pMAL-p5× (p for periplasmic expression), which encodesMBP-ACE2NTD fusion with predicted molecular mass (MW) of ∼94.0 kDa (mature protein had 26-aa signal peptide removed from MBP N-terminus, 44.5 + 49.5 = 94.0 kDa). The first six aa residues (MAVTAA) serve as a short linker between MBP and ACE2NTD.

SARS-CoV-2 His-tagged Spike (S) protein, the receptor binding domain (RBD) protein of S and recombinant ACE2 protein were purchased from Sino Biological (Beijing/China and Wayne/United States).

### Construction of ACE2NTD-GFP Fusion

The coding sequence for ACE2NTD was amplified by PCR and inserted into p DasherGFP (*Nde*I cut, pBR322 backbone, Amp^R^) (source reference: atum.bio). GFP expression was under the control of T5 promoter and is inducible by addition of IPTG. GFP-expressing colonies, GFP, and ACE2NTD-GFP protein bands were visualized under long UV light or by a fluorescence imager (Typhoon, GE Lifesciences) at 520 nM (Cy2 channel, 515–535 nm emission).

### Cloning of *Escherichia coli* RNase I Coding Sequence Into pET21b With a C-Terminal 6×His Tag and Construction of RNase I-ACE2NTD Fusion

The RNase I gene (rna) was amplified from *E. coli* genomic DNA (*E. coli* strain H709c) and assembled into T7 expression vector pET21b (*Nde*I and *Xho*I digested), which produced a C-terminal 6×His tag. The protein expression level was evaluated in four T7 expression strains: T7 Express (C2566, NEB), T7 Express with LysY and *lacI*^*q*^ (C3013, NEB), T7 SHuffle (C3026, *E. coli* K strain, NEB), and Nico (λDE3) with reduced histidine-rich background proteins (NEB). The RNase I precursor contains a signal peptide (amino acid residues 1–23) for export to periplasm. To create RNase I-ACE2NTD fusion, a PCR fragment amplified from ACE2NTD gene block (IDT) was assembled into the *Xho*I site of pET21b-RNase I, which generated the coding sequence for the fusion RNase I-ACE2NTD (6×His). The entire gene was sequenced using T7 universal primer (S1248), T7 terminator primer (reverse) (S1271), and internal ACE2 primers. The expression of RNase I-ACE2NTD (6×His) fusion was evaluated in five T7 expression strains: C2566, C3013, C3026, Nico (λDE3), and BL21 (λDE3). RNase I (6×His) was partially purified by chromatography through Ni-NTA column (Ni-NTA agarose fast-flow, Thermo Fisher Scientific). The protein was concentrated by low-speed centrifugation in an Amicon concentrator (10 kDa cut-off) and resuspended in a storage buffer SB (50 mM Tris–HCl, pH 7.5, 200 mM NaCl, 10 mM DTT, 50% glycerol).

### Cloning of *Escherichia coli* RNase III Gene (rnc) Into pET21b With a C-terminal 6×His Tag

The RNase III gene (rnc) was amplified from *E. coli* genomic DNA (*E. coli* strain H709c) and assembled into T7 expression vector pET21b (*Nde*I and *Xho*I digested), which produced a C-terminal 6×His tag. The protein expression level was evaluated in three T7 expression strains: T7 Express with LysY and *lacI*^*q*^ (C3013), T7 SHuffle (C3026, *E. coli* K strain), and Nico (λDE3). RNase III (6×His) was partially purified by chromatography through Ni-NTA column (Ni-NTA agarose fast-flow, Thermo Fisher). The protein was concentrated by low-speed centrifugation in a concentrator and resuspended in a storage buffer SB. RNase III activity was assayed using a 40 mer duplex RNA and dsRNA ladder (NEB).

### Cloning of *Escherichia coli* RNase I (No Native Signal Peptide Coding Sequence) Gene Into pMAL-p5×

A PCR fragment encoding *E. coli* RNase I (GenBank Sequence ID: AAB40811.1) was inserted into pMAL-p5× to generate the following construct (the 23-aa signal sequence of RNase I was deleted). The molecular weight of the fusion protein MBP-RNase I is predicted to be ∼71.7 kDa (44.5 + 27.2 kDa).

(P_tac_)-MBP-(*Nde*I)-

(MKAFWRNAALLAVSLLPFSSANA) LALQAKQYGDFDRYVLALSWQTGFCQSQHDRNRNERD

ECRLQTETTNKADFLTVHGLWPGLPKSVAARGVDERRWMRFGCATRPIPNLPEARASRMC

SSPETGLSLETAAKLSEVMPGAGGRSCLERYEYAKHGACFGFDPDAYFGTMVRLNQEIKE

SEAGKFLADNYGKTVSRRDFDAAFAKSWGKENVKAVKLTCQGNPAYLTEIQISIKADAIN

APLSANSFLPQPHPGNCGKTFVIDKAGY (*Bam*HI).

### Cloning of Human RNase A Gene (Human Rnase1) and Construction of hRNaseA-ACE2NTD150 Fusion

Two versions of fusion were constructed: fusion 1 contains the *E. coli* MBP signal peptide (aa residues 1–26; this signal peptide directed the fusion protein to be exported to the periplasmic space) and fusion 2 without the MBP signal peptide. The fusion consisted of peptides: *E. coli* MBP signal peptide (MBP residues 1–26) + hRNase A (hRNase A residues 29–156) + ACE2NTD (ACE2 N-terminal residues 18–150, referred to as ACE2NTD150) (note: the aa residue numbers refer to the native protein amino acid number).

MKIKTGARIL ALSALTTMMF SASALA (*E. coli* MBP signal peptide).

KESRAKKFQRQHMDSDSSPSSSSTYCNQMMRRRNMTQGR CKPVNTFVHEPLVDVQNVCFQEKVTCKNGQGNCYKSNSS MHITDCRLTNGSRYPNCAYRTSPKERHIIVACEGSPYVPVHF DASVEDST (hRNase A)

GSAGSA (artificial aa linker)

QSTIEEQAKTFLDKFNHEAEDLFYQSSLASWNYNTNITEENVQNMNNAGDKWSAFLKEQSTLAQMYPLQEIQNLTVKLQLQALQQNGSSVLSEDKSKRLNTILNTMSTIYSTGKVCNPDNPQECLLLEPGHHHHHH (ACE2NTD150-6×His).

The predicted molecular mass of hRNaseA-ACE2 (6×His) with and without MBP signal peptide is 33.3 and 30.7 kDa, respectively. The fusion gene blocks were purchased from IDT and assembled into T7 expression vector pET21b (*Nde*I and *Xho*I cut), which resulted in a C-terminal 6×His tag. The protein expression level was evaluated in three T7 expression strains: T7 Express with LysY and *lacI*^*q*^ (C3013), T7 SHuffle (C3029, *E. coli* B strain), and Nico (λDE3, C2529H). Soluble hRNaseA-ACE2 (6×His) fusion without signal peptide was purified from 1 L of IPTG-induced cells (cells induced at 16°C overnight) by chromatography through a Ni-NTA agarose column or from 3 ml of cell lysate (30 ml of IPTG-induced cells) by binding to Ni magnetic beads.

### RNase I and RNase III Activity Assays

RNase III assay: 1 μg of double-stranded (ds) RNA substrates were digested with either ShortCut^®^ RNase III (NEB) or RNase III (6×His) for 15 min at 37°C in a total volume of 15 μl of 1 × reaction buffer (50 mM Tris–HCl, 1 mM DTT, 50 mM NaCl, pH 7.5) supplemented with 20 mM MnCl_2_, and stopped by addition EDTA (50 mM). Cleavage products were analyzed by gel electrophoresis on a 2% agarose gel.

Single-strand (ss) RNA, double-strand (ds) RNA ladders, and 5′ fluorescein-labeled RNA (300 nt) were provided by NEB and used as substrates for RNase I and RNase III activity assays. Cleavage products were analyzed by gel electrophoresis on PAGE-Urea gel or agarose gel. A 5′ FAM-labeled SARS-C0V-2 RNA (50 mer) was purchased from IDT (synthetic RNA sequence:

5′ FAM/rArUrGrGrArGrArGrCrCrUrUrGrUrCrCrCrUrGr GrUrUrUrCrArArCrGrArGrArArArArCrArCrArCrGrUrCrCr ArArCrUrCrArG 3′, RNase-free, HPLC purified). RNase I assay: 16 nM of the RNA substrate was digested by purified MBP-RNase I, RNase I (6×His), or refolded RNase I-ACE2NTD (6×His) in a high-salt buffer (NEB buffer 3: 100 mM NaCl, 50 mM Tris–HCl, pH 7.9 at 25°C, 10 mM MgCl_2_, 1 mM DTT) or a high-salt buffer without divalent cations (100 mM NaCl, 50 mM Tris–HCl, pH 7.5, 1 mM DTT) as RNase I is active in the absence of divalent cations or in the presence of EDTA (Bechhofer and Deutscher, 2019). Cleavage reactions were quenched by eliminating enzymes with the treatment with Proteinase K (1.6 U, NEB), and the products were analyzed by capillary gel electrophoresis (CE assay). Substrate and cleavage product peaks were analyzed by PeakScan software (ABI/Thermo Fisher).

### Protein Pull-Down Assays (Protein Complex Pull-Down by Ni Magnetic Beads)

*Ni-magnetic beads pull down of His-tagged S protein in complex with MBP-ACE2NTD.* Ni-magnetic beads (100 μl) were washed three times with Ni binding buffer (50 mM NaH_2_PO_4_, 0.3 M NaCl, 20 mM imidazole). His-tagged Spike protein (2 μg, purchased from Sino Biological) was added to the beads pre-suspended in 200 μl of Ni binding buffer and incubated at 4°C in a roller for 30 min. Refolded MBP-ACE2NTD protein (10 μg) or equal volume of storage buffer was added to the prebound Ni-beads/S protein and co-incubation continued at 4°C for 1 h. The Ni magnetic beads were washed three times with Ni binding buffer (1 ml each time), and the protein was eluted by addition of 50 μl of Ni elution buffer (50 mM NaH_2_PO_4_, 0.3 M NaCl, 0.25 M imidazole). Approximately 17 μl of the eluted protein was loaded onto 10–20% SDS-PAG gel for analysis.

### Amylose Magnetic Beads Pull Down of His-Tagged S Protein and RBD Protein

NEB’s protocol for MBP protein binding to the amylose magnetic beads was used with minor modification. Amylose magnetic beads (400 μl) in an Eppendorf tube were saturated (blocked) in 1 ml of BSA (1×) in MBP binding buffer (200 mM NaCl, 20 mM Tris–HCl, pH 7.4, 1 mM EDTA, 1 mM DTT) on a rotator at 4°C overnight. The beads were then washed three times (15 min each) at 4°C with MBP binding buffer. The magnetic beads were resuspended in 200 μl of MBP binding buffer. His-tagged SARS-CoV-2 Spike protein (5 μl) or His-tagged RBD protein (5 μl at 1.75 μg/μl, purchased from Sino Biological) was first diluted in 45 μl of MBP binding buffer. MBP-ACE2 fusion protein (25 μl at 0.98 μg/μl) was mixed with diluted Spike protein or RBD protein and 100 μl of BSA-saturated magnetic beads. Protein binding (complex formation) was carried out on a rotator at 4°C overnight. The beads with bound proteins were washed three times with 1 ml of MBP binding buffer at 4°C for 15 min. Bound proteins were eluted with the addition of 50 μl of MBP elution buffer (MBP binding buffer plus 10 mM maltose), and the eluted proteins were analyzed by Western blot using anti-His Ab or anti-ACE2 Ab.

### Western Blot Using Anti-6×His Antibody (Ab) and Mouse Monoclonal Anti-ACE2 Ab

Mouse monoclonal anti-ACE2 Ab (catalog number #74512) and mouse anti-His tag Ab (catalog number 70796-3) were purchased from Cell Signaling Technologies (CST, MA) and EMC Millipore (Germany), respectively. Protein expression, purification, and interaction between protein partners were monitored by Western blot analysis according to the manufacturer’s protocol. Briefly, cell extract or purified proteins (6×His tag) were subjected to 10–20% SDS PAGE, and proteins were transferred onto Protran BA85 nitrocellulose (Whatman) in a 0.5 × transfer buffer. The transferred proteins were stained with Ponceau S solution (Sigma-Aldrich). The nitrocellulose membrane was soaked (blocked) in 3% milk (dry milk from Bio-Rad) solution in 1 × PBS following by incubation with appropriate primary antibody solution (1:1,000 dilution) for 1 h at room temperature. The excess primary antibodies were washed off with 1 × PBS containing 0.1% Tween 20 and 0.1% Triton X100 three times for 15 min, followed by incubation with secondary anti-mouse IgG HPR-conjugated antibodies (1:1,000) (CST) and secondary Ab, washed three times with 1 × PBS, 0.1% Tween20 and 0.1% Triton X100. Signal detection was performed by treatment with LumiGlo reagent (CST) and visualized by fluorescent imaging.

## Results

### Expression and Purification of Maltose-Binding Protein-Angiotensin-Converting Enzyme 2 N-terminal Domain Fusion Protein

**FIGURE 1:**
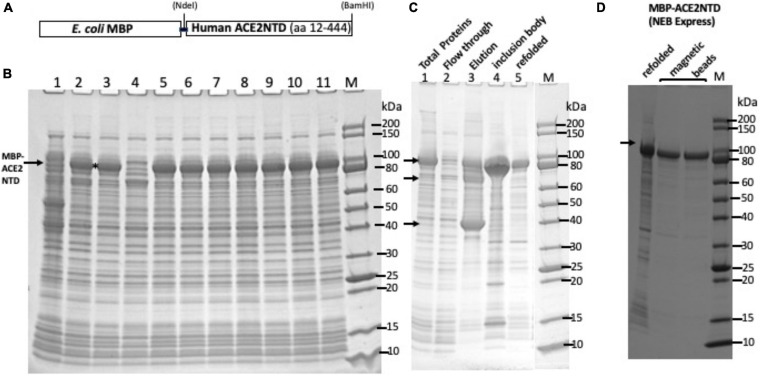
SDS-PAGE analysis of maltose binding protein-angiotensin converting enzyme 2 N-terminal domain (MBP-ACE2NTD) fusion. **(A)** A schematic diagram of the fusion protein. **(B)** Total cellular proteins from IPTG-induced cells (NEB Express) containing the MBP-ACE2NTD fusion. Nine out of 11 clones tested here expressed the fusion (except #1 and #4). Clone #8 was chosen for further study. **(C)** MBP-ACE2NTD fusion expressed from IPTG induced cells (T7 Shuffle, lanes 1–4). Lane 1, total cellular protein; lane 2, flow-through from an amylose column; lane 3, eluted MBP-ACE2NTD protein from an amylose column; lane 4, MBP-ACE2NTD protein found in the pellet (inclusion body); lane 5, refolded MBP-ACE2NTD fusion protein from cell pellet (NEB Express); M, protein size marker (10–200 kDa, NEB). Arrows indicate the MBP-ACE2NTD protein, a truncated protein, and host MBP. **(D)** Purified MBP-ACE2NTD protein by amylose magnetic beads. Lane 1, refolded fusion; lanes 2 and 3, protein eluted in a Tris–HCl buffer (20 mM, pH 7.5) and sodium phosphate buffer (0.1 M, pH 8.0) with 10 mM maltose.

A schematic diagram of the fusion consisting of an N-terminal MBP and a C-terminal ACE2NTD as well as the cloning sites is shown in Figure 1A. Total cellular proteins from sonicated cell lysate of IPTG-induced cells (NEB Express, 16°C overnight) were analyzed by SDS-PAGE (Figure 1B). Nine out of 11 clones produced a strong protein band of ∼94 kDa, close to the predicted size. Clone #8 contains the correct sequence and was chosen for further analysis. After 15 min of centrifugation of the total lysate in a microcentrifuge, however, most of the MBP-ACE2NTD fusion was found in the pellet (inclusion body) (data not shown). To improve protein folding and solubility, NEB SHuffle *E. coli* host was tested for protein induction. This strain was engineered to promote disulfide bond formation in the cytoplasm and is deficient in proteases Lon and OmpT. One liter of cell culture was induced with IPTG at 16°C overnight, and the supernatant of cell lysates (the soluble fraction) was loaded onto a 15-ml amylose column. Figure 1C shows the partially purified fusion protein using an amylose column (∼6 mg protein purified from 1 Lof IPTG-induced cells including a few contaminating proteins). The ∼40-kDa protein is likely to be the host MBP without the signal peptide. To remove this major contaminating protein, an MBP-deficient SHuffle strain will be required. Some fusion proteins expressed in NEB SHuffle strain were found in the pellet (Figure 1C, lane 4). The protein pellet from NEB Express cells was solubilized. After solubilization and refolding, the fusion protein was partially purified (Figure 1C, lane 5). The protein yield from solubilized pellet (NEB Express) was approximately 50 mg/L of IPTG-induced cells. The refolded protein can be further purified by incubation with amylose magnetic beads (Figure 1D). It was concluded that most of the fusion protein made in NEB Express was found in the pellet (inclusion body). One liter of IPTG-induced NEB SHuffle cells produced ∼6 mg of soluble protein and ∼50 mg of insoluble protein in the pellet. The production of MBP-ACE2NTD protein from *E. coli* cells may have a cost advantage over ACE2 protein produced from human cell cultures, but the ACE2NTD from bacterial source lacks the glycosylation compared with the human version.

### Maltose-Binding Protein-Angiotensin-Converting Enzyme 2 N-terminal Domain Binding to Spike (S) Protein and Receptor Binding Domain (RBD) of S Protein

One potential application of MBP-ACE2NTD protein is to serve as a decoy to bind SARS-CoV-2 in which MBP can bind to immobilized amylose beads. We examined MBP-ACE2NTD binding to S and RBD protein *in vitro* by protein pull-down assay. The His-tagged S protein is expected to bind to the Ni magnetic beads. The MBP-ACE2NTD protein was incubated with the S protein pre-bound to the Ni magnetic beads. After extensive washing with high salt buffer, the S protein and potential bound partner were eluted with an elution buffer. In a control experiment, approximately 20% of the S protein can be recovered from the Ni magnetic beads (Supplementary Figure 1A, compared lanes 1 – input and lane 2 – recovered). When the S protein was incubated with MBP-ACE2NTD protein, only a very weak band of the binding partner was recovered, indicating poor binding of S protein to MBP-ACE2NTD *in vitro* under the high-salt condition (0.3 M NaCl). In a reversed pull-down assay, the MBP-ACE2NTD bound to the amylose magnetic beads was incubated with S or RBD proteins. After extensive washing, the proteins were eluted with a maltose buffer. Only a weak band of the S protein was detected by anti-6×His Ab in a Western blot (Supplementary Figure 1B). The RBD protein binds to the binding partner modestly well, as detected by anti-His Ab in the Western blot. In the control experiment, MBP-ACE2NTD was detected by monoclonal anti-ACE2 mAb. The protein binding and pull-down results indicate that the SARS-Cov-2 S protein binds poorly to the MBP-ACE2NTD protein, and the RBD protein binds reasonably well to the fusion *in vitro*. This result is consistent with published data that the SARS-CoV-2 S protein, unlike SARS-CoV-1 S protein, is a poor binder to the human ACE2 receptor *in vitro* (Lubbe et al., 2020; Wrapp et al., 2020; Yan et al., 2020). It remains to be seen whether some recent SARS-CoV-2 variants (e.g., E484K, N501Y, P681H, and ΔH69/ΔV70 variants, P.1 variant with K417T/E484K/N501Y amino acid changes in S protein) with higher transmission rate can bind to the ACE2 receptor protein more tightly *in vitro* (P.1 variant reference^2^) (Kemp et al., 2020).

### ACE2NTD-GFP Fusion

Since ACE2NTD fusion to MBP displayed reduced solubility, it is anticipated that fusion of ACE2NTD to GFP may reduce green fluorescence. ACE2NTD-GFP fusion was constructed by insertion of ACE2NTD coding sequence into the GFP expression vector, creating N-terminal ACE2NTD and C-terminal GFP. GFP-expressing *E. coli* cells (IPTG induced) showed strong green fluorescence and formed green colonies under long UV light or at 520-nm light emission (Supplementary Figure 2A). The ACE2NTD-GFP expression cells (IPTG-induced NEB Express and T7 Express), however, did not show green fluorescence; presumably, the ACE2NTD-GFP fusion negatively affected the protein folding and/or expression inside cells. IPTG-induced expression of ACE2NTD-GFP fusion showed a weak green fluorescence in NEB SHuffle cells, suggesting improvement of folding of the fusion protein. To further confirm this, the total proteins from IPTG induced cells were analyzed by SDS-PAGE, and protein bands were visualized on a fluorescence imager. GFP showed a strong fluorescence signal, but the ACE2NTD-GFP fusion protein showed a weak signal in NEB SHuffle K and B strains (Supplementary Figure 2B) (the fluorescence signal presumably resulted from protein refolding after washing three times in Milli-Q water). It was concluded that ACE2NTD likely negatively affected the folding and/or expression of GFP when they were fused together. This fusion system may be used to isolate ACE2NTD variants with improved folding and stronger fluorescence in *E. coli* from ACE2NTD mutant libraries by simply screening for green colonies among thousands of transformants. It has been shown before that membrane protein fusion to GFP can create fusion problem for GFP with reduced fluorescence (Alguel et al., 2010). GFP-expressing cell suspensions and lysate (IPTG-induced) showed a yellow/green color under normal light (Supplementary Figures 2C,D).

### Expression of MBP-RNase A (Bovine) and MBP-RNase I Fusions

**FIGURE 2:**
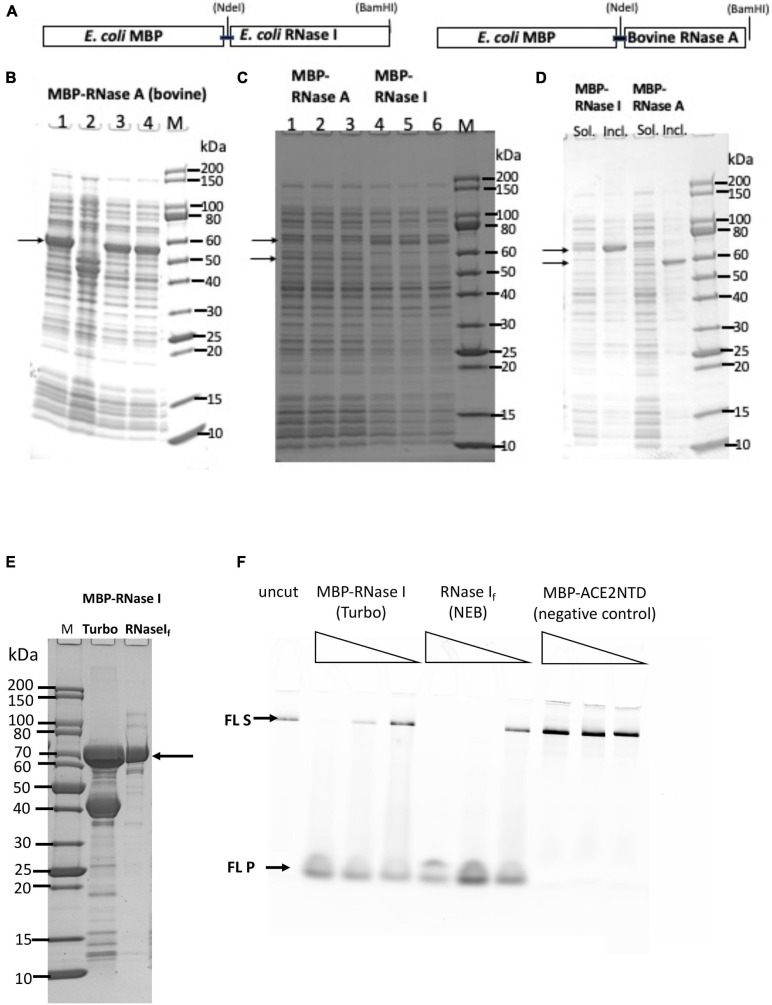
SDS-PAGE analysis of MBP-RNase I and MBP-RNase A fusions. **(A)** Schematic diagram of MBP-RNase fusion. **(B)** Total cellular proteins from IPTG-induced cells (NEB Express). **(C)** Supernatant (soluble) of the fusion proteins (three independent isolates each). Arrows indicate the fusions. **(D)** Comparison of supernatant (Sol.) and pellet (Incl.) of the fusions. M, protein size marker (NEB). **(E)** SDS-PAGE analysis of partially purified MBP-RNase I fusion. Lane 1, protein size marker; lane 2, MBP-RNase I fusion purified from IPTG-induced NEB Turbo cells (C2984); lane 3, RNase I_f_ (NEB). **(F)** RNase activity on fluorescein-labeled RNA (300 nt). No enzyme (uncut) and MBP-ACE2NTD fusion serve as negative controls. S, substrate; P, products.

A schematic diagram of the *E. coli* RNase I and bovine RNase A fusion with MBP is shown in Figure 2A. The fusion proteins should be exported to the periplasm and the MBP signal peptide being cleaved, but some fusion proteins could exist in the cytoplasm due to the protein over-expression and overwhelming of the export system. Analysis of four cell lysates (total proteins) from NEB Express indicated a strong induced band of 55 to 60 kDa in three out of four samples, in close agreement with the predicted MBP-RNase A fusion (58.3 kDa) (Figure 2B). The inserts in plasmid clones (#1, #3, and #4) were sequenced to confirm the correct DNA sequence. After centrifugation, most of the fusion protein was pelleted, and a small fraction was found in the supernatant (Figures 2C,D). Attempt to transfer the MBP-RNase A expression clone into NEB SHuffle strain was not successful (few colonies were found in transformation, data not shown), suggesting the fusion protein may be toxic to the host. Bovine RNase A is a single-strand specific enzyme, but at high concentrations, it can cleave dsRNA as well (Raines, 1998). Its over-expression in T7 SHuffle cells appeared to be lethal to the host. A signal peptide may be added to bovine RNase A to be exported to the periplasm for its over-expression in *E. coli*. Due to the low yield of soluble MBP-RNase A (bovine) fusion, the purification was not pursued further.

A PCR fragment encoding the *E. coli* RNase I was inserted into the pMAL-p5× vector to construct MBP-RNase I fusion. Analysis of 11 cell lysates (total proteins) from IPTG-induced cells (NEB Express) indicated a strong induced band of ∼70 kDa in eight out of 11 samples, in agreement with the predicted MW (71.7 kDa) (Figure 2C and Supplementary Figure 3). After centrifugation, most of the fusion protein was found in the pellet, and a small fraction remained in the supernatant (Figures 2C,D). Attempt to express the fusion in T7SHuffle cells did not improve the expression level (data not shown). MBP-RNase I fusion appeared to be toxic to T7 SHuffle host since only low-density cells could be obtained in liquid culture at 37°C. The protein yield of MBP-RNase I was highest in NEB Turbo cells, probably due to the robust cell growth and higher cell weight per liter culture. Figure 2E shows the partially purified MBP-RNase I fusion in comparison with RNase I_f_ (RNase I_f_ = MBP-RNase I fusion from NEB as a research grade ribonuclease, M0243S). The enzyme preparation also contained the host MBP. In order to eliminate the MBP, it will be necessary to use an MBP-deficient strain as the expression host. In a ribonuclease activity assay, the partially purified MBP-RNase I is active in the digestion of an RNase substrate (fluorescein-labeled 300 nt RNA) (Figure 2F).

Previous studies showed that *E. coli* RNase I does not require divalent cations for nuclease activity, and the other eight ribonucleases from *E. coli* are only active in the presence of divalent cations (Bechhofer and Deutscher, 2019). To demonstrate the ribonuclease activity for the partially purified MBP-RNase I, we incubated a low range ssRNA ladder (N0364S, NEB) with the enzyme (three enzyme dilutions at 2, 0.2, and 0.02 μg) in a high-salt buffer supplemented with EDTA, Ni^2+^, Ca^2+^, Mn^2+^, Co^2+^, or Mg^2+^. MBP-RNase I is most active in Ca^2+^, Mn^2+^, and Co^2+^ buffers. It is also active in EDTA, Ni^2+^, and Mg^2+^ buffers, but with limited digestion at low enzyme input (Supplementary Figure 4A). It is possible that in Ca^2+^, Mn^2+^, and Co^2+^ buffers, MBP-RNase I activity is stimulated at a low enzyme concentration. It was concluded that MBP-RNase I fusion can be expressed at a moderate level in NEB Turbo and NEB Express cells. A small fraction of the fusion protein was found in the soluble fraction and can be purified by chromatography through amylose column. The partially purified fusion is active in digestion of RNA in the absence of divalent cations or in the presence of EDTA.

### Expression and Purification of *E. coli* RNase I (6×His)

**FIGURE 3:**
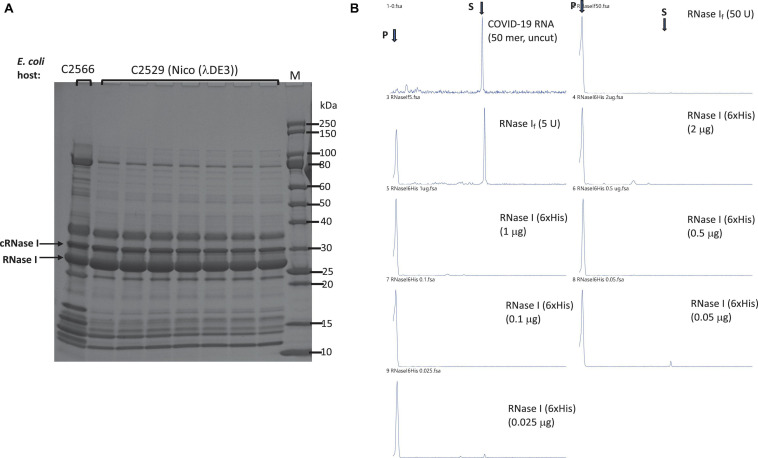
Purification of RNase I (6×His) and RNase activity assay. **(A)** Purification of RNase I (6×His) from Nickel-NTA agarose column. Lane 1, RNase I (6×His) pooled fractions from a nickel column (purified from T7 Express cell extract). Arrows indicate the cytoplasmic RNase I precursor (cRNase I) with the signal peptide (predicted MW 30.7 kDa), and the periplasmic RNase I with the signal peptide removed (predicted MW 27.0 kDa). **(B)** RNase activity on a FAM-labeled SARS-CoV-2 RNA (50 mer). S = substrate; P = cleavage product(s). Positive controls, 50 and 5 U of RNase I_f_ (NEB). RNase I (6×His) enzyme titration (2 μg to 25 ng protein) was used in the activity assay to digest fixed amount of RNA (16 nM) in NEB buffer 3 at 37°C for 1 h. Proteinase K (1.6 U) was added to remove RNase I. The final cleavage products were analyzed by capillary electrophoresis (CE), and peaks were visualized by PeakScan.

To improve the expression level of RNase I, we also expressed aC-terminal 6×His-tagged version. *E. coli rna* gene was inserted into the T7 expression vector pET21b. Itsexpression level was compared with two T7 expression strains (T7 Express and Nico (λDE3). *E. coli* RNase I has its own signal peptide to be exported to the periplasm, but we detected two versions in over-expression: RNase I precursor with the signal peptide in cytoplasm and the mature enzyme without the signal peptide. The proteins eluted from C2529 cell lysate contained fewer contaminating proteins than C2566 cells, due to the knock-out mutations of a few Histidine-rich proteins in C2529 (Figure 3A). Two versions of RNase I appeared to be co-purified: a longer cytoplasmic version (cRNase I) with the signal peptide (precursor, 30 kDa), and the shorter version of the periplasmic form (27 kDa). The partially purified enzyme is active in the digestion of FAM-labeled COVID-19 RNA (50 mer) in a high-salt buffer in the absence of MgCl_2_ (Figure 3B). We also tested the ribonuclease activity in the presence and absence of divalent cations. RNase I (6×His) is active in the presence of 10 mM EDTA. The RNase activity is mostly active in buffers with Ca^2+^, Mn^2+^, or Co^2+^ at low enzyme concentration (data not shown). In Ca^2+^ buffer, *E. coli* RNase I also displays ribonuclease activity on dsRNA (Grünberg et al., 2021).

### Expression and Purification of *Escherichia coli* RNase III (6×His)

**FIGURE 4:**
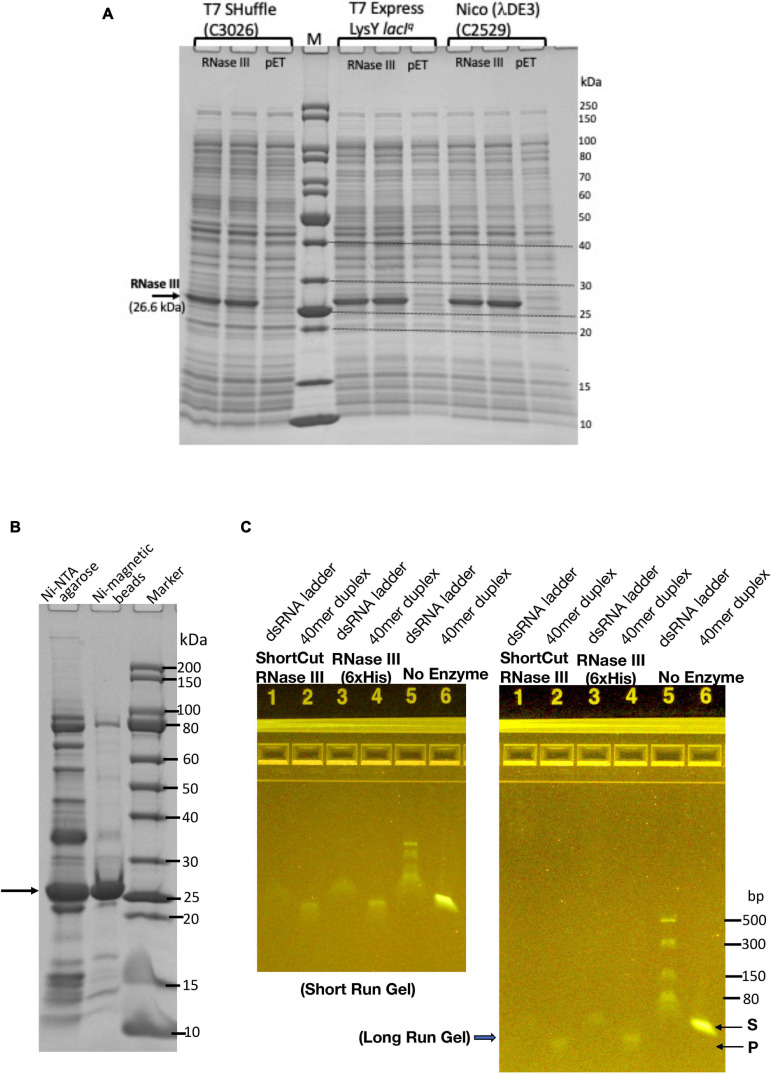
Expression and purification of *E. coli* RNase III and RNase activity assay on dsRNA. **(A)** RNase III expression level in three *E. coli* T7 strains: T7 Shuffle (C3026, K strain), T7 Express with *lacI*^*q*^ and LysY (C3013), and Nico (λDE3). **(B)** Purified RNase III from nickel-NTA agarose column chromatography and Ni magnetic beads. **(C)** Ribonuclease activity assay on dsRNA (40 mer duplex and dsRNA ladder).

Since the SARS-CoV-2 RNA genome also contains regions of dsRNA (e.g., RNA hairpin stem loops structures in the 5′ untranslated region and frame shift element) (Andrews et al., 2020; Miao et al., 2021). RNase III may be needed to digest the duplex RNA regions. The *E. coli rnc* gene encoding RNase III was cloned into pET21b to generate a C-terminal 6×His-tagged protein. RNase III expression level after IPTG induction was examined in three T7 expression strains: T7 SHuffle (C3026, K strain), T7 Express with LysY and *lacI*^*q*^ (C3013), and Nico (λDE3) (C2529). Figure 4A shows the expression levels (soluble fractions/supernatant) in the three strains. RNase III (6×His) was partially purified from a Nickel-NTA agarose column or Ni magnetic beads (Figure 4B). It is active in digestion of a 40 mer RNA duplex and dsRNA ladder. The 40 mer duplex RNA and dsRNA ladder were digested into smaller fragments as detected in the agarose gel (Figure 4C). In a control experiment, the ShortCut RNase III (MBP-RNase III fusion) also digested the RNA substrates and gave rise to similar shortened fragments. It was concluded that RNase III (6×His) can be expressed in *E. coli* at a high level, and it is not toxic to *E. coli* T7 expression strains. The expression level is comparable with the three strains tested.

### Expression and Purification of RNase I-ACE2 (6×His) Fusion Protein

**FIGURE 5:**
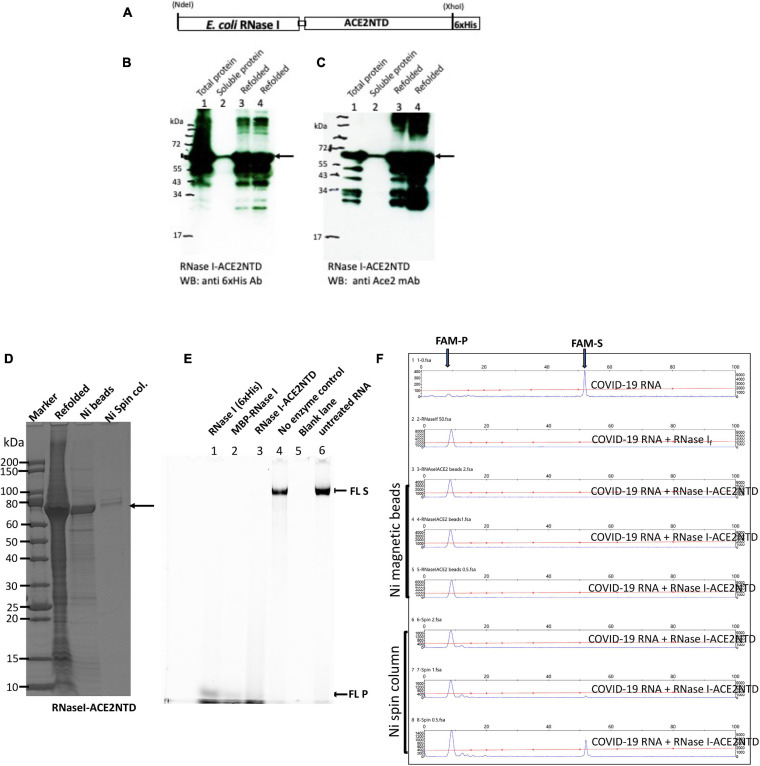
SDS-PAGE and Western blot analysis of RNase I-ACE2NTD fusion and activity assays. **(A)** Schematic diagram of RNase I-ACENTD (6×His) fusion. **(B)** Western blot analysis of RNase I-ACE2NTD in total protein, supernatant (soluble), and refolded protein using anti-His mAb. **(C)** Same as in **(B)**, except using anti-ACE2 mAb. **(D)** SDS-PAGE analysis of the refolded RNase I-ACE2NTD fusion and further purified protein by Ni magnetic beads and Ni spin column. **(E)** RNase I-ACE2NTD (refolded) ribonuclease activity on fluorescein (FL)-labeled RNA (300 nt) in NEB buffer 3. RNase I (6×His) and MBP-RNase I were used as positive controls. **(F)** Ribonuclease activity of RNase I-ACE2NTD (purified by Ni magnetic beads or Ni spin column) on SARS-CoV-2 RNA (50 mer). RNase I_f_, a positive control. FAM-S, FAM-labeled substrate; FAM-P, FAM labeled cleavage product(s).

Since our constructs of MBP-ACE2NTD and RNases were shown to be folded and soluble, we next sought to generate the fusion protein that can bind S protein and exhibit cleavage activity. The RNase I-ACE2NTD (6×His) fusion was expressed in Nico (λDE3). Most of the fusion was found in the inclusion body and only a small fraction was found in the supernatant as detected by anti-6×His Ab or anti-ACE2 monoclonal Ab in Western blot and SDS-PAGE (Figures 5A–C and Supplementary Figure 5). The RNase I-ACE2NTD (6×His) fusion pellet was subjected to a refolding procedure and further purified by binding to Ni magnetic beads or Ni spin column (Figure 5D). The refolded fusion protein is active in the digestion of a 300-nt RNA substrate (Figure 5E), similar to the positive controls RNase I (6×His) and MBP-RNase I. The fusion enzyme purified through Ni magnetic beads and spin column is also active in the digestion of COVID-19 RNA (60 mer) (Figure 5F). We have not tested the ribonuclease activity on full-length COVID-19 RNA due to local bio-safety regulation on the virus.

### Expression and Purification of hRNase A-ACE2NTD150 Fusion With and Without Export Signal Peptide

**FIGURE 6:**
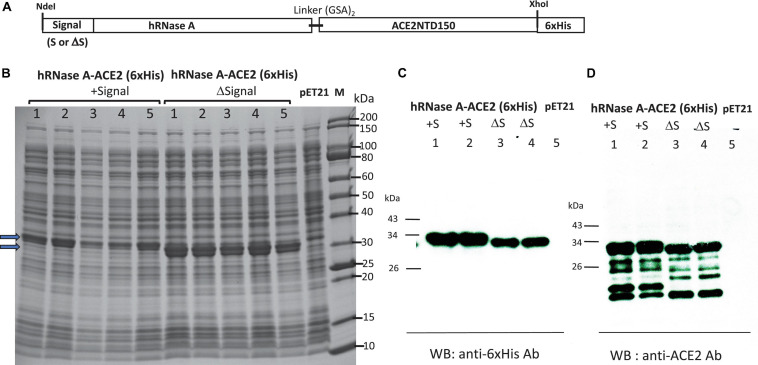
Expression of hRNase A-ACE2NTD150 in T7 Express LysY/*lacI*^*q*^ (C3013). **(A)** Schematic diagram of the fusion protein. **(B)** SDS-PAGE analysis of five isolates of hRNase A-ACE2NTD150 (five isolates with MBP signal peptide, five clones without MBP signal peptide). Total cell lysate of pET21b serves as a negative control. **(C,D)** Western blot analysis of the fusion proteins (soluble fraction/supernatant) by anti-His mAb and anti-ACE2 mAb.

A schematic diagram of fusion of hRNase A with ACE2NTD150 (a shorter version of ACE2NTD) is shown in Figure 6A. A short linker (GSAGSA) is inserted between the fusion partners. hRNase A’s native signal peptide was deleted, and MBP signal peptide was added. The second clone does not carry any signal peptide (cytoplasmic version). The over-expressed fusions could be detected in IPTG-induced cell lysate (total proteins) as shown in Figure 6B. The two fusions appeared as closely migrated doublet. It may have something to do with multiple disulfide bonds in hRNase A (eight Cys residues in total, potentially forming four disulfide bonds). With protein loading dye, DTT and heating before SDS-PAGE, some of the disulfide bonds may be reduced to –SH groups, creating a mixture of isoforms with different secondary structures (S–S vs. –SH). Another possibility is that export systems are overwhelmed, and some of the protein with signal peptide is not secreted. To confirm the identity of the engineered fusion, we performed Western blot using mouse anti-His Ab and anti-ACE2 monoclonal Ab. Figure 6C shows the soluble fusion proteins detected by the antibodies. There are some minor degradation products from the fusions as detected by the anti-ACE2 mAb (Figure 6D). We also tested fusion expression level in Nico (λDE3) and NEB SHuffle T7 (B strain). hRNase A-ACE2NTD150 fusions with or without the MBP signal peptide can be over-expressed in Nico (λDE3) cells (data not shown). However, hRNase A-ACE2NTD150 fusion expression is toxic to the Shuffle strain: hRNase A-ACE2NTD150 with signal peptide formed small colonies in the transformation of T7 SHuffle. No transformants were found with plasmid carrying the coding sequence for hRNase A-ACE2NTD150 (ΔS, lacking MBP signal peptide). This result is similar to the toxic effect by MBP-RNase A (bovine) in the transformation of T7 SHuffle (K strain).

**FIGURE 7:**
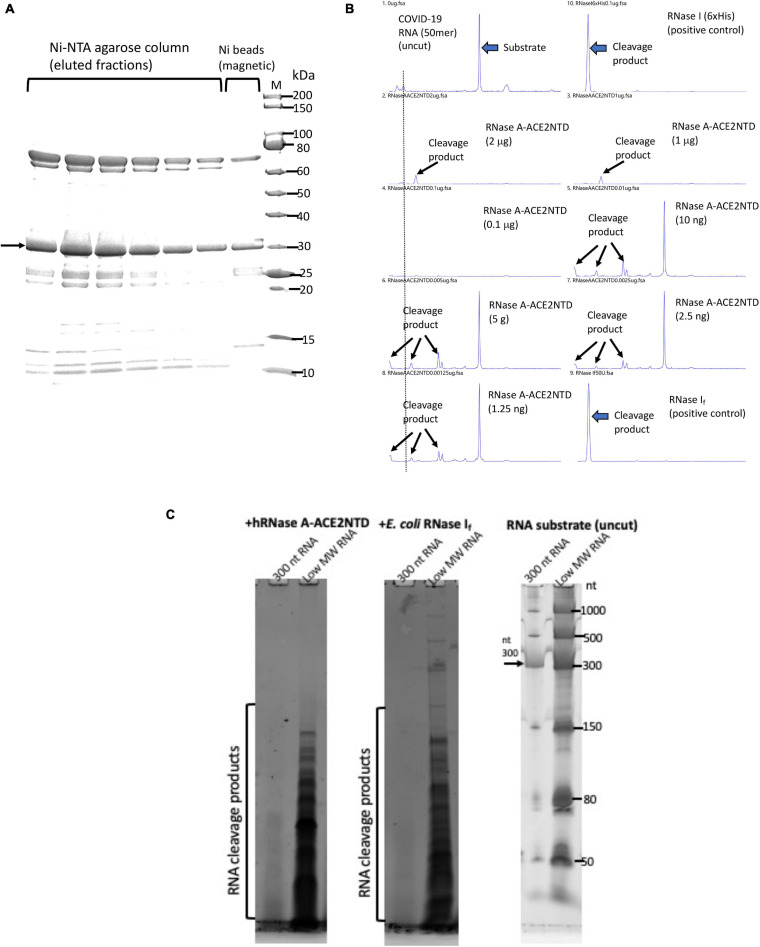
SDS-PAGE analysis of purified hRNase A-ACE2NTD150 (6×His) and ribonuclease activity assays. **(A)** Partially purified hRNase A-ACE2NTD150 (6×His) (no signal peptide) by Ni-NTA agarose column chromatography or by binding to Ni magnetic beads. **(B)** RNase activity on FAM-labeled SARS-CoV-2 RNA (50 mer). Arrows indicate the substrate and cleavage products in the CE assay. **(C)** RNase activity assay on a 300-nt RNA and low MW RNA ladder as analyzed on a 6% PAG-urea gel (stained with SYBR green and visualized on a Typhoon Imager).

Most of the hRNase A-ACE2NTD150 fusions (with or without MBP signal peptide) were found in the pellet following centrifugation (data not shown). However, there are still some soluble fusions in the supernatant. The fusion protein hRNase A-ACE2NTD150 (6×His) (no signal peptide) was partially purified by chromatography through a Ni-NTA agarose column or binding to Ni magnetic beads (Figure 7A). The purified fusion proteins were active in cleaving FAM-labeled COVID-19 RNA (50 mer) (Figure 7B). The substrate RNA completely disappeared after 30 min of digestion (at 0.1 to 2 μg of the fusion protein). Both RNA substrates and cleavage products were detected after digestion at a lower enzyme concentration (at 1.25 to 10 ng) (Figure 7B). Some smaller cleavage products less than 10 nt were not easily detected in the CE assay. To further confirm the ribonuclease activity, we digested a 300-nt RNA and an ssRNA ladder (low MW). Both RNA substrates were degraded by the fusion enzyme as detected on a 6% PAG-urea gel and staining with SYBR green (Figure 7C). It was concluded that the fusion enzyme hRNase A-ACE2NTD150 displays ribonuclease activity on both 50 mer COVID-19 RNA and other RNA substrates ranging from 50 to 1,000 nt. We have not tested ribonuclease activity on the full-length SARS-CoV-2 viral RNA. The binding affinity to RBD domain of the Spike protein remains to be tested.

The fusion protein expression and purification conditions and activity assays on RNA substrates are summarized in Table 1.

**TABLE 1:** *Escherichia coli* expression strains, purification method, and enzyme activity/potential usage.

Protein construct	*E. coli* host	Purification method	Protein function or RNase activity
MBP-ACE2NTD^a^	T7 SHuffle or NEB Express	Amylose column or protein refolding, amylose magnetic beads	Binding to S-RBD
MBP-RNase I^b^	NEB Turbo or NEB Express	Amylose column	Cleaving 300-nt RNA
ACE2NTD-GFP	T7 Shuffle	Not determined (ND)	Low green fluorescence
RNase I (6×His)	T7 Express (LysY/*lacI*^*q*^) or Nico (λDE3)	Ni-NTA agarose column	Cleaving RNA ladder and 50 mer COVID-19 RNA
RNase III (6×His)	T7 Express (LysY/*lacI*^*q*^) or Nico (λDE3)	Ni-NTA agarose column	Cleaving duplex RNA and dsRNA ladder
RNase I-ACE2NTD (6×His)	Nico (λDE3) or BL21 (λDE3)	Protein refolding and Ni magnetic beads	Cleaving 300-ntRNA and 50 mer COVID-19 RNA
hRNase A-ACE2NTD150 (6xHis)^c^	Nico (λDE3) or T7 Express (LysY/*lacI*^*q*^)	Ni-NTA agarose, Ni magnetic beads	Cleaving COVID-19 RNA and ssRNA ladder

*^a^ACE2 signal peptide removed.*

*^b^Native RNase I signal peptide removed.*

*^c^Human RNase A original signal peptide removed.*

## Discussion

This work demonstrated that MBP-ACE2NTD, MBP-RNase I, RNase I (6×His), RNase III (6×His), RNase I-ACE2NTD (6×His), and hRNase A-ACE2NTD150 can be expressed in *E. coli* either as a soluble protein or refolded protein from inclusion bodies. We tested a number of *E. coli* expression strains and found that MBP-ACE2NTD expression was partially soluble in T7 SHuffle strain, while RNase I-ACE2NTD (6×His) fusion was best expressed in Nico (λDE3) or BL21 (λDE3) as a refolded protein from inclusion body. RNase I (6×His) and RNase III (6×His) expressions were more comparable among the T7 expression strains tested in this work. The over-expression of MBP-RNase A (bovine) hRNase A-ACE2NTD150 fusions, however, is toxic to T7 SHuffle strains. We conclude from this work that different *E. coli* expression strains will need to be tested in order to optimize the expression conditions. We only tested low temperature induction (16 to 18°C), and the protein induction level at higher temperatures has not been examined. It is anticipated that protein expression level may be increased due to a higher cell weight at a higher temperature with possible trade-off of lower protein solubility. Large-scale fermentation and low-cost purification will be required for mass production and use of these enzymes as anti-viral reagents. Other protein expression systems, for example, the yeast expression system, could be used to over-express human ACE2 receptor and TMPRSS2. In addition, human ACE2 receptor protein and the serine protease can be expressed and exported to the surface of bacteria and yeast for cell surface display (Klemm and Schembri, 2000). Homing endonuclease variants have been expressed and selected successfully in yeast surface display; the engineered endonuclease mutants displayed altered specificities with a range of cleavage activities *in vitro* (Jacoby et al., 2012). The fusion of ACE2NTD to *E. coli* FimH protein to display on the cell surface did not generate positive results in our preliminary study: the fusion coding sequence can be constructed in DNA level, but no fusion protein was detected by anti-His mAb to detect FimH signal peptide-ACE2NTD-FimH (6×His) fusion. Presumably the fusion is toxic to the host cell.

The ACE2NTD-GFP fusion may be used as a bio-sensor if more soluble ACE2NTD variants could be isolated. The tight binding of COVID-19 variants to the ACE2NTD-GFP fusion may alter the fluorescence signal intensity.

Future research may be directed toward making full-length recombinant ACE2 receptor from *E. coli* with co-expression of chaperone proteins to increase solubility (Chaperone plasmid set, Takara Bio) (Gopal and Kumar, 2013). In a previous work, we found that co-expression of *E. coli* GroEL/ES proteins with GmrSD endonuclease greatly improved the solubility of GmrSD (He et al., 2015). To reduce protein purification cost, expression of RNase I-ACE2 (6×His) and hRNase A-ACE2 (6×His) fusions may be carried out using the *K. lactis* yeast expression system (NEB) to export the recombinant proteins into the culture medium for industrial production of the fusions.

Human RNase A, bacterial RNase I and III, or RNase-ACE2 fusion can degrade viral RNA if one can find conditions to destabilize virus particles, triggering viral RNA release and degradation. Proteinase K treatment of SARS-CoV-2 clinical samples to release viral RNA for RT-qPCR has been routinely adopted in diagnostic procedures. Thermolabile Proteinase K treatment, heat inactivation of the protease, and further digestion by RNase I or RNase A can significantly reduce the copy number of pseudovirus RNA genome. The use of mild detergent, EDTA, heating, and non-toxic chemicals may also destabilize the infectious virus particles and partial release of viral RNA for nuclease destruction. The enzymatic and chemical approaches can be complementary under different environmental settings. More research is needed to validate the enzymatic approach to reduce virus counts and transmission in air droplets and to combat highly transmissible COVID-19 variants.

It is noted that the experiments described in this work were carried out with small fragments of RNA *in vitro* and that work with a real virus is needed to fully evaluate the effectiveness as an anti-viral strategy.

## Data Availability Statement

The original contributions presented in the study are included in the article/Supplementary Material, further inquiries can be directed to the corresponding author.

## Author Contributions

S-YX conceived the idea. S-YX, AF, T-HC, and EY performed the experiments and analyzed data. S-YX wrote the manuscript with editing help from AF, T-HC, and EY. All authors contributed to the article and approved the submitted version.

## Conflict of Interest

S-YX, AF, T-HC, and EY are employees of New England Biolabs, Inc., New England Biolabs is a manufacturer and vendor of molecular biology reagents, including several enzymes and buffers used in this study. This affiliation does not affect the authors’ impartiality, adherence to journal standards and policies, or availability of data. The authors declare that this study received funding from New England Biolabs, Inc., The funder was not involved in the study design, collection, analysis, interpretation of data, and the writing of this article. The funder encouraged to submit this work for publication.

## Footnotes

1 www.neb.com

2 https://virological.org/t/sars-cov-2-reinfection-by-the-new-variant-of-concern-voc-p-1-in-amazonas-brazil/596
