# Supplementary material for: Expression of Human ACE2 N-terminal Domain, Part of the Receptor for SARS-CoV-2, in Fusion With Maltose-Binding Protein, E. coli Ribonuclease I and Human RNase A
Source: Front Microbiol. 2021 Jun 11;12:660149. doi: 10.3389/fmicb.2021.660149 (PMC8226257; doi:10.3389/fmicb.2021.660149)
Supplement: Supplementary file 1 [file Data_Sheet_1.docx]

**Supplementary Material: Suppl. Fig. S1-S5.**

**Expression of human ACE2 N-terminal domain, part of the receptor for SARS-CoV-2, in fusion with maltose binding protein, *E. coli* ribonuclease I and human RNase A**

Shuang-yong Xu*, Alexey Fomenkov, Tien-Hao Chen, and Erbay Yigit

New England Biolabs, Inc. 240 County Road, Ipswich, MA 01938, USA

*Correspondence:

E-mail: [xus@neb.com](mailto:xus@neb.com)

Telephone: 1-978-380-7287

key words: human ACE2 receptor, *E. coli* ribonuclease I (RNase I), RNase III, RNase I-ACE2NTD fusion, human RNase A-ACE2NTD fusion

**Supplementary figures.**

**Suppl. Fig. S1. Protein pull-down assays for MBP-ACE2NTD, Spike (S) and RBD protein. A.** His-tagged Spike protein bound to Ni magnetic beads incubated with MBP-ACE2NTD and co-eluted proteins. **B.** MBP-ACE2NTD bound to amylose magnetic beads incubated with Spike or RBD protein and co-eluted proteins as detected by anti-His and anti-ACE2 mAbs.

**
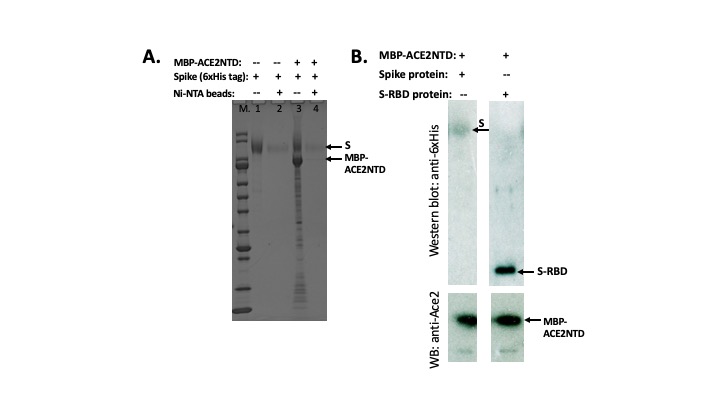
**

**Suppl. Fig. S2. Green fluorescence detection for GFP and ACE2NTD-GFP fusion protein. A.** T7 Express (LysY/*lacI^q^*) cells carrying pBR322 (negative control) and pBR322-P_T5_-*lacO*-DasherGFP visualized under long UV light. **B.** *E. coli* colonies (Amp^R^ transformants) of NEB SHuffle and T7 Express cells expressing GFP or ACE2NTD-GFP fusion visualized under long UV light. GFP expressing colonies show bright green color. T7 Express [ACE2NTD-GFP] shows no green fluorescence. NEB SHuffle [ACE2NTD-GFP] shows a weak green fluorescence. **C.** Total proteins from cell lysates of NEB SHuffle [ACE2NTD-GFP] (K and B strains) detected by fluorescence imaging at 520 nm (Cy2 channel). Arrows indicate GFP and ACE2NTD-GFP fusion, respectively. The Protein ladder shows no fluorescence and was not detected in the imager. **D.** Cells suspension of C3013 [pBR322] and C3013 [GFP]. **E.** T7 Express (C2566) cell lysates of ACE2NTD-GFP fusion and GFP. GFP expressing cells and cell lysate show yellow/green color under normal light as visualized by naked eyes.

**
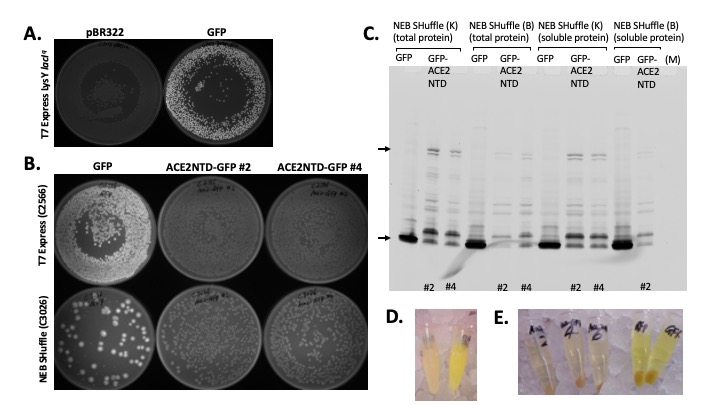
**

**Suppl. Fig. S3. Comparison of protein expression in three *E. coli* strains: NEB Turbo (C2983), NEB Express (C2523), NEB T7 SHuffle (C3026, K strain).** MBP-ACE2NTD (ACE), MBP-TMPRSS2 (PRS, lacking the transmembrane domain), MBP-RNase I (RI), MBP-RNase A (RA). **A.** SDS-PAGE analysis of total proteins in cell lysate. **B.** SDS-PAGE analysis of soluble proteins (supernatant) in cell lysate. “*” indicates the expected target protein.

**
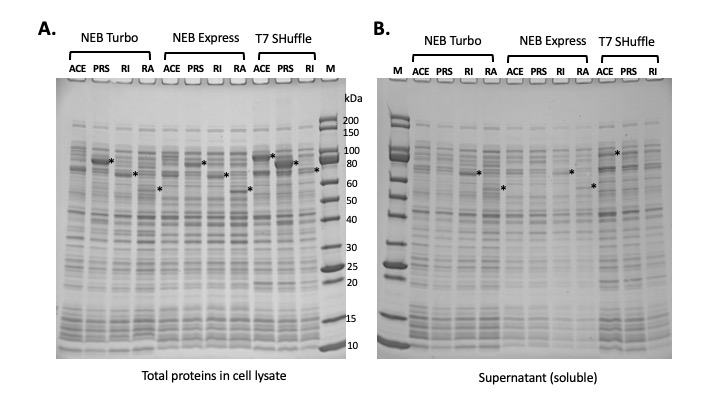
**

**Suppl. Fig. S4. Ribonuclease activity in the presence of EDTA and divalent cations. A.** MBP-RNase I ribonuclease activity assay. The low-range RNA ladder (50 to 1000 nt long, NEB) was used as the substrate for RNase activity assay in a high sale buffer (100 mM NaCl, 50 mM Tris-HCl, pH 7.5) supplemented with divalent cations (1 mM) or EDTA (10 mM). RNase I does not require divalent cations for nuclease activity. It is active in the presence of EDTA (1 to 10 mM).


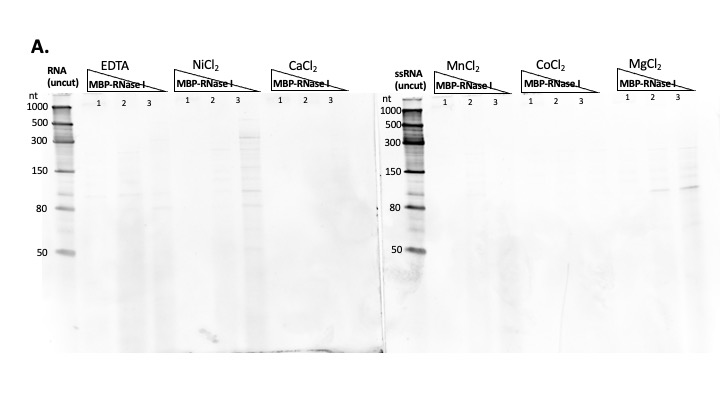


**Suppl. Fig. S5. SDS-PAGE and Western blot analysis of *E. coli* lysate expressing RNase I (6xHis), RNase III (6xHis), and RNase I-ACE2NTD (6xHis) fusion proteins. A.** Total proteins in cell lysate. **B.** Soluble proteins in the supernatant after centrifugation in a microcentrifuge (10k rmp/min, 15 min). **C** and **D**. 6xHis-tagged proteins detected by anti-His mAb in a Western blot. Arrow and “*” indicate the target proteins. Color prestained protein marker (not shown) was from NEB.

**
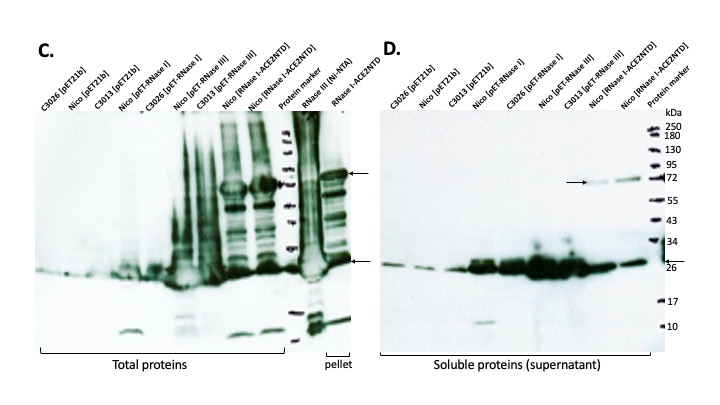
**
